# Optimal Span between Feet of Public Squat Toilet Based on Anthropometric Data and Squatting Stability Assessment

**DOI:** 10.3390/healthcare9010042

**Published:** 2021-01-05

**Authors:** Yi-Lang Chen, Resy Kumala Sari, Ying-Hua Liao, Wei-Cheng Lin

**Affiliations:** 1 Department of Industrial Engineering and Management, Ming Chi University of Technology, New Taipei 24301, Taiwan; resy_kumalasari@universitaspahlawan.ac.id (R.K.S.); m07218007@mail2.mcut.edu.tw (Y.-H.L.); m07218003@mail2.mcut.edu.tw (W.-C.L.); 2 Department of Industrial Design, Chang Gung University, Touyuan 33302, Taiwan; 3 Program Study of Industrial Engineering, Universitas Pahlawan Tuanku Tambusai, Riau 28412, Indonesia

**Keywords:** squatting-type toilet, span between feet (SBF), stability, comfort

## Abstract

Sitting toilets are preferred globally because they afford a relatively comfortable posture. However, squat toilets are among the most common toilets in numerous public areas because of their advantages, including personal hygiene, easy cleaning, and health benefits. This study attempted to determine optimal toilet design parameters and recruited 50 Taiwanese and 50 Southeast Asian women and collected span between feet (SBF) data for participants squatting in their most comfortable posture, and also surveyed maximum outer width (MOW) data of 28 public squat toilets in Taipei. Finally, we compared the squatting stability levels of 40 female participants (20 Taiwanese and 20 Southeast Asians) who squatted for 2 min at comfortable SBF and MOW-based SBF values. The results revealed that the minimum and maximum SBFs of Taiwanese were 14.52 cm and 18.40 cm, and that of Southeast Asians were 15.64 cm and 20.40 cm, respectively. No significant difference was observed in the SBFs between the two groups was observed. The mean (range) MOW of the surveyed toilets was 27.7 (27–29) cm. Analysis of variance results showed no difference in stability between the two SBFs. This implies that the comfortable SBF (i.e., 16 cm between the participants’ heels) was narrower than the MOW, as commonly used, indicating that the comfortable SBF can be considered as an optimal toilet width parameter because of its constant stability.

## 1. Introduction

Defecation posture as well as its effect on defecation and urination has been examined for numerous years. Sitting toilets symbolize modernization and have been adopted in nearly all countries globally. By contrast, squat toilets, which prevent humans from being polluted by feces and save effort in defecation, constitute the most basic toilet style [1,2]. Thus, whether the traditional squatting position for defecation is superior to the more evolved sitting posture adopted in Western cultures has stimulated intense debate in both the East and the West [3].

Investigators have promoted the squatting posture owing to its health benefits from a physiological perspective [4,5,6,7,8]. Specifically, in the squatting posture, the anorectal angle is widened (100–110°) and the rectum is straightened, resulting in smoother defecation [2]. Moreover, squat toilets can protect users from colon and prostate diseases [9,10] and help in reducing the occurrence of diseases related to the digestive system, such as constipation and hemorrhoids [11]. For pregnant women, the squatting posture is considered superior to the sitting posture because it prevents pressure from being applied to the uterus; daily squatting also helps prepare them for a more natural delivery [12]. In addition, squat toilets are ecofriendly because they consume less water than do other toilets [13].

A large-scale survey demonstrated that many women tended to use public squat toilets, although most of the respondents reported using sitting toilets at home [14]. A field survey from Taiwan also revealed that most respondents (86%) agreed that public squat toilets more effectively satisfied their sanitary requirements compared with other toilet types [15]. Specifically, they used the squatting posture more frequently than they did the sitting posture when away from home. This explains why most public toilets, particularly in many Asian countries, are equipped with two typical toilet types (i.e., squat and sitting) to enable users to choose according to their habits [15,16]. In addition, Yu [2] indicated that some house toilets in old cities in several Asian countries are usually squat toilet types. Public toilets are available in all places, including parks, shopping centers, transportation centers, gas stations, and educational institutes [17].

In recent years, many Southeast Asian women have visited China and Taiwan for nursing or field work and have become more adaptive to the public squat toilets than Westerners. This is because Asians frequently use squatting postures in their daily work activities as well as when defecating. Moreover, Asians are generally considered to be proficient at the so-called Asian squat, which means that they can completely touch the ground with their feet when squatting [18], although no formal research data are available to confirm this consideration.

Scholars have reported that the squatting position is a nonneutral leg posture, which could lead to lower extremity injuries. Specifically, studies have shown that the squatting position can create discomfort in the back, legs, and even the whole body [19,20,21,22]. Tucker et al. [23] investigated the squatting position and revealed that the maximum squatting distance can deteriorate body balance. Furthermore, research revealed that prolonged squatting/stooping postures can elicit back and leg fatigue, which may compromise standing stability and balance [24]. This implies that stability and comfort constitute another crucial issue associated with the use of squat toilets. However, considering the high frequency of peoples’ daily use of toilets, relatively little attention has been paid to the evaluations in both stability and comfort of squatting toilet use. Even though manufacturers are currently developing toilets exhibiting high efficiency, especially in terms of cleanliness and aesthetics, they rarely consider the design from the ergonomics perspective. In the very few ergonomic studies on squatting toilets, Cai and You [15] conducted a test to examine the effects of the span between feet (SBF) and footstep slope on squatting comfort and observed that a 15° slope was most preferred by participants. They also developed the new squat toilet prototypes based on these participants’ SBF values and footstep slope. However, they recruited only 71 men and nine women. Regarding physiological characteristics between genders, women tend to have a higher demand for squat toilets than do men, especially when urinating. Therefore, squatting toilet designs based on female use are definitely contributive.

An integral ergonomic concept is to fit the product to the people. On the basis of this concept, the present study attempted to determine optimal design parameters for squat toilets by collecting SBF data for participants squatting in their most subjectively comfortable posture. In addition, this study determined differences in squatting stability levels between the measured SBF and maximum outer width (MOW) data measured for public toilets. Finally, on the basis of these measurement results, a new concept of squat toilet design was proposed.

## 2. Materials and Methods

This study involved three stages. In the first stage, 100 female participants in Taipei were randomly recruited; for SBF data collection, the participants were requested to assume their most comfortable squatting posture. In the second stage, public toilets in Taipei were surveyed. In the third stage, 40 female participants were recruited and underwent a stability test under two SBF conditions in order to compare their squatting stability levels between these conditions. The Ethics Committee of National Taiwan University approved our study protocol and all study participants provided written consent prior to the experiment.

### 2.1. Stage 1: SBF Measurement during Squatting

**Figure 1:**
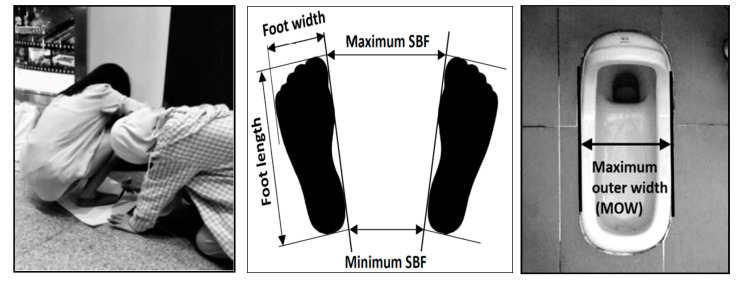
Measurements of the span between feet (SBF) and maximum outer width (MOW) of public squat toilets.

SBF measurement was conducted with the participants in their most comfortable squatting posture, and the measured data were used to assess whether the current SBF in public squat toilets is appropriate. In this stage, 100 female participants were randomly recruited (50 Taiwanese, constituting one group; and 50 Southeast Asians, constituting another group) for SBF measurement. The mean (standard deviation, SD) age, height, body weight, and body mass index (BMI) of the participants were 22.8 (3.8) years, 158.4 (6.2) cm, 52.9 (8.7) kg, and 21.1 (3.3), respectively. The mean (SD) foot length and foot width of the participants were 23.1 (1.5) and 10.1 (1.0) cm, respectively. During the measurement, the participants were requested to squat without wearing shoes or slippers on a preset large paper (52 cm × 38 cm) placed on flat ground. Each participant was encouraged to squat down (i.e., Asian squat) and adjust her feet position and body posture to the most comfortable position based on her perceptions. Any participant who could not successfully executed the Asian squat was excluded from the sample. Once the participant confirmed the position, the experimenter marked the contours of her feet on the paper, which included foot length, foot width, and minimum and maximum SBF (i.e., min_ and max_SBF, Figure 1), for further measurement according to the procedure described by Cai and You [15].

### 2.2. Stage 2: MOW Measurement of Public Squat Toilets

Twenty-eight public squat toilets in Taipei were surveyed in this stage. Toilets in schools, train stations, MRT stations, parks, and gas stations were surveyed to collect MOW data for further analysis in stage 3. The MOW measurement procedure is illustrated in Figure 1.

### 2.3. Stage 3: SBF Measurement during Squatting

In this stage, 40 female participants (20 Taiwanese and 20 Southeast Asians), all of whom were healthy and physically active, were recruited and underwent a stability test. Their mean (SD) age, height, body weight, and BMI were 23.6 (1.9) years, 158.7 (6.0) cm, 52.9 (6.82) kg, and 21.0 (2.0), respectively. The mean (SD) foot length and width of the Taiwanese participants were 23.4 (0.8) and 9.7 (0.8) cm, respectively, and those of the Southeast Asian participants were 23.4 (1.6) and 9.9 (0.7) cm. All participants were required to possess a healthy physique, especially in the lower limbs, and to be conversant with squat toilets. A Biodex balance system (BBS, 950-440, Biodex Medical Systems Inc., Shirley, NY, USA) was used to evaluate who-body stability levels for participants squatting on the platform under various test conditions. The BBS was applied in accordance with the procedures described by Minoonejad et al. [25] and Khan et al. [26].

**Figure 2:**
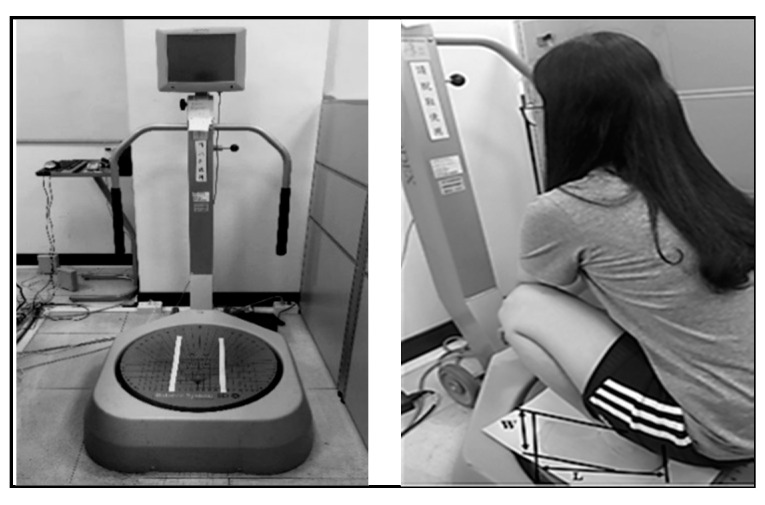
Measurement of squatting stability on the Biodex balance system platform.

Before the stability test, the platform was calibrated to 0°, and each participant was guided according to the standard procedure to familiarize themselves with the test before the experiment (Figure 2). The test evaluated the squatting stability of each participant under two SBF conditions: One of the conditions involved the participants’ most comfortable SBF (stage 1), and the other involved the SBF based on the MOW of the public squat toilets (stage 2). Each participant applied the two SBFs in random order. The test objective was to assess whether current public squat toilets must be improved on the basis of stability levels of participants in the most comfortable squatting position. The stability measurement was executed under the “static” mode in the BBS, meaning that the platform was in the most stable state. This mode was set because the test was used to simulate squatting postures during defecation; each test trial was sustained for 2 min, with a 10-min rest interval between trials. The 2-min testing period was determined on the basis of our pilot test, which revealed 2 min to be the maximum period for which all participants could maintain their posture without feeling any discomfort. Huang [16] also revealed that under a normal use condition for public portable toilets, Taiwanese females tended to defecate and urinate within 103 and 94 s, respectively, on average.

During the stability test, the participants were requested to squat and open their eyes, without holding any support structures such as walls, machine holders, or the floor. They were instructed to maintain their arms in their natural positions, and they did not receive semantic feedback during the test. The BBS indicated four quadrants (anterior, posterior, left, and right) on the green line on the model plate, which indicated body weight shifts. The results were recorded as overall stability index (OSI) scores calculated using a previously proposed equation [27]. A higher OSI score was considered to indicate less stability and greater postural variability in balancing the body on the platform. According to Desseauve et al. [28], the participants adopted the Asian squat (with the feet flat) on the platform.

### 2.4. Statistical Analysis

The data collected from stages 1 and 2 were analyzed using descriptive statistics (i.e., mean, SD, and independent *t*-test). A Pearson product-moment correlation (r) was also used to examine the relationships between the participants’ SBFs and anthropometric data (i.e., participants’ height, body weight, foot length, and foot width). Data obtained from stage 3 were analyzed using a two-way analysis of variance (ANOVA) to assess the effect of the two SBFs on squatting stability. The independent variables were region (Taiwan and Southeast Asia) and SBF (comfortable SBF and SBF based on MOW). The dependent variable was the OSI during the 2-min squatting period. All statistical analyses were conducted using statistical software (SPSS 22.0, IBM Corp., Armork, NY, USA) with the level of significance being set at 0.05.

## 3. Results

### 3.1. SBF Measurement

The SBF data obtained for 100 participants are presented in Table 1. The mean (SD) min_ and max_SBF values for the Taiwanese participants were 14.52 (5.6) and 18.40 (7.3) cm, respectively, and those for the Southeast Asian participants were 15.64 (4.5) and 20.40 (7.0) cm, respectively. However, the basic data (i.e., age, height, body weight, BMI, and foot size) of the participants revealed no significant difference between the two groups, by using an independent t test. Regardless of the region variable, no significant correlation was observed between the SBFs and anthropometric data (all *p* > 0.05). Although the Southeast Asian participants’ SBF values were a little higher than those of the Taiwanese participants, the values did not differ significantly between the groups according to the t test (Table 1). In the analysis, we set a min_SBF of 16 cm and max_SBF of 20 cm as comfortable SBFs in the stability test.

### 3.2. MOW of Public Squat Toilets

In the MOW measurement, we mainly collected MOW data for the sampled public squat toilets in Taipei. We analyzed 28 squat toilets, and the mean MOW was 27.7 cm, with a small range of 27–29 cm. ALEX was the most popular squat brand (46.4%), followed by HCG (35.7%). We observed that the MOW values were relatively consistent, indicating that the squat toilets equipped in the public areas were somewhat standardized in their size. On the basis of the mean MOW, we set the SBF to 28 cm for further stability analysis.

### 3.3. Squatting Stability Test

Table 2 presents the mean (SD) of OSI values for the independent and dependent variables. The mean (SD) OSI values for the Taiwanese group were 18.3 (6.8) and 17.8 (5.7) for the most comfortable and MOW conditions, respectively, and the corresponding values for the Southeast Asian group were 12.7 (2.9) and 13.5 (2.9). Table 3 shows the two-way ANOVA results. The region variable significantly affected the OSI, whereas the SBF and region × SBF did not affect the OSI. Moreover, the Southeast Asian group exhibited more stability than did the Taiwanese group when squatting under the same SBF conditions during a testing period of 2 min.

## 4. Discussion

According to the results of the study, the min_SBF and max_SBF values derived for the Taiwanese participants were 14.52 and 18.40 cm, respectively, and those derived for the Southeast Asian participants were 15.64 and 20.40 cm, respectively. These results are inconsistent with those obtained by Cai and You [15], who reported min_SBF and max_SBF values of 16.6 and 32.2 cm, respectively. However, the difference in SBFs exists obviously in max_SBF. The substantial difference in max_SBF values between the two studies is attributable to the sex difference (e.g., height, hip width, and muscle strength of lower limbs), which may influence the squatting posture. Cai and You included 71 men and nine women for analysis; by contrast, all participants were women in our study. Compared with max SBF, min SBF is more crucial for determining the contour of a newly designed toilet. Ideal scenarios for the use of min_SBF 16 cm would be those that involve shape optimization, weight reduction, and fabrication cost reduction.

In the analysis, when each participant’s foot length (i.e., mean 23.1 cm) was considered and transferred by min_SBF and max_SBF, each of the participant’s feet was transversely inclined outward at an angle of approximately 9°; that is, the outward angle between the feet was 18°. This angle is twice that obtained by Cai and You [15], which was approximately 36°. However, Cai and You recruited 80 participants and assessed their preference and physical load among four footstep slopes (i.e., 0°, 15°, 30°, and 45°); they observed that the 15° slope was rated by the participants to be the most preferable and caused the least heart rate increase. The slope is close to that revealed in our study. As presented in Table 1, we observed no difference in min_SBF or max_SBF between the two groups (all *p* > 0.05), indicating no difference in squatting comfort between these two groups of participants. This also indicates that a new toilet can fit the need of these two user groups.

In stability analyses, a higher OSI typically indicates considerable movement (i.e., considerable instability) during a squatting test with static measures [29]. We observed that the mean OSI values of the Taiwanese participants were 18.3 and 17.8 when the SBFs were set to 16 and 28 cm, respectively, and that those of the Southeast Asian participants were 12.7 and 13.5 at these two SBFs, respectively (Table 2). The two-way ANOVA revealed that the region variable significantly affected the OSI, whereas the SBF and region × SBF did not affect the OSI. These results were as expected and reasonable. According to our SBF assessment results, no difference in squatting stability existed between the two SBF conditions.

Regarding the SBF value of squatting posture, the participants’ squatting comfort and stability may be unrelated. In the survey conducted by Cai and You [15], among women using squat toilets, 66% reported leg numbness and only 19% reported imbalance in posture. This also demonstrates that although the difference in SBF was up to 12 cm in our test, no significant difference in stability existed between the two SBF conditions. Accordingly, the MOW value of 28 cm determined for the public squat toilets in Taipei may not be a golden rule, although nearly all public squat toilets follow this design parameter. That is, at the same stability level, an SBF of 16 cm was more comfortable for squatting than that of 28 cm. Squatting in an uncomfortable position may result in pain in the entire body, upper and lower legs, and lower back [19,20,21,22]. Thus, we can reasonably infer that users squatting at an SBF of 16 cm can maintain their postural stability as observed at an SBF of 28 cm, and have the most comfortable posture.

The region variable had a significant effect on squatting stability (Table 3). The Southeast Asian participants exhibited a more stable squatting posture than did the Taiwanese participants under the same SBF conditions during a 2-min testing period. Additionally, there was no significant difference in feet size between these two groups. A possible reason for the difference in OSI is that the Southeast Asian participants more commonly assumed the squatting posture in their daily activities than did the Taiwanese participants. This suggests that any new squat toilet designed for Taiwanese women would also be appropriate for Southeast Asian women.

**Figure 3:**
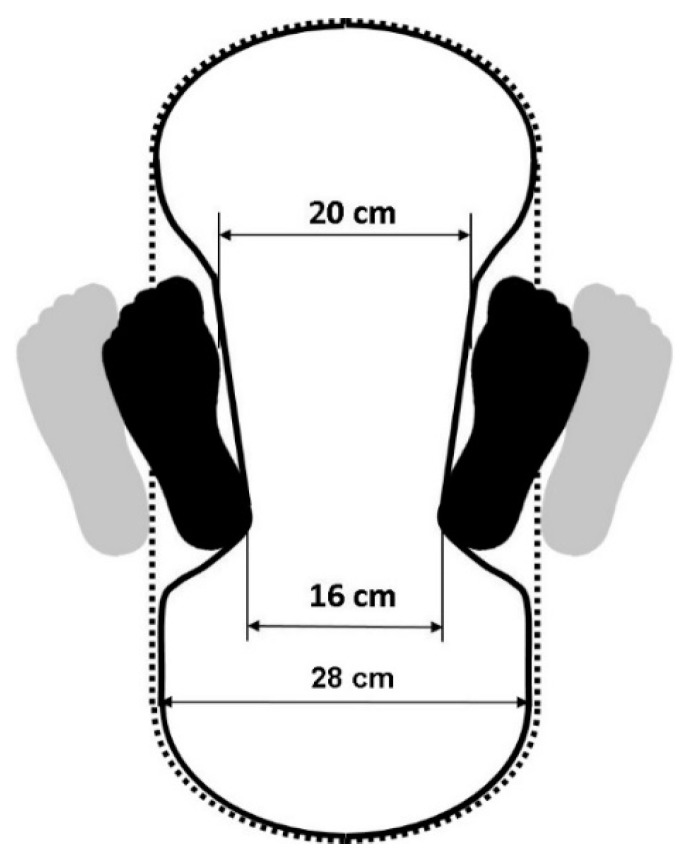
Remodeled squat toilet with a peanut- or dumbbell-shaped design (solid line: a new design in the study; dotted line: a typical squat toilet).

Modern squat toilets have been designed on the basis of traditional squat toilets and from an ergonomic perspective, including a streamlined smooth outer shape; this has considerably enhanced users’ comfort [2]. As mentioned, because of the MOW measured for public toilets, an SBF of 16 cm is more comfortable than that of 28 cm. This narrower SBF could thus constitute the primary reference for the concept of the new toilet design. Accordingly, this study proposed a new toilet model (Figure 3) primarily exhibiting a peanut- or dumbbell-shaped design and having a relatively narrow SBF. However, experimental anthropometric data constituted the basis of our proposed toilet design. This study suggests that existing public squat toilets could be redesigned to improve the squatting comfort. However, this newly designed toilet also accepts the users to adopt a wider SBF for their experienced comfort.

This study has some limitations. We used a 2-min testing period for squatting stability assessment, which is shorter than that in a previous study [15], and neglected worse cases involving a longer defecation time. In addition, we conducted the min_ and max_SBF measurement processes on bare-footed participants. Although this approach could control the influence of shoes, the measurement condition differed slightly from actual toilet conditions. These limitations should be considered when applying the results of this study.

## 5. Conclusions

The primary finding of the study is that no difference in SBF was observed between the two regional groups, and no difference in squatting stability between the two SBF conditions (i.e., 16 and 28 cm). The most comfortable SBF was 16 cm; this SBF can be considered as a parameter for new toilet width designs. This study thus proposed a new squat toilet model with a peanut- or dumbbell-shaped design.

## Figures and Tables

**Table 1:** Mean (standard deviation, SD) span between feet (SBF) values of two participant groups and the independent *t*-test results.

Regions	*N*	Minimum_SBF (cm)		Maximum_SBF (cm)	
		Mean	SD	Mean	SD
Taiwanese	50	14.52	5.64	18.40	7.32
Southeast Asians	50	15.64	4.54	20.40	6.99
Total	100	15.08	5.09	19.40	7.16
Difference		t = −1.096, *p* = 0.212		t = −1.402, *p* = 0.953	

**Table 2:** Mean (standard deviation; SD) of overall stability index based on two span between feet (SBF) levels (Comfortable _SBF vs. SBF by maximum outer width [MOW]).

Regions	N	Comfortable SBF		SBF by MOW	
		Mean	SD	Mean	SD
Taiwanese	20	18.3	6.8	17.8	5.7
Southeast Asians	20	12.7	2.9	13.5	2.9
Total	40	15.5	5.9	15.6	5.0

Note: SBF-span between feet.

**Table 3:** Two-way analysis of variance result for overall stability index values.

Variables	SS	DF	MS	F	*p*
Region	483.4	1	483.4	20.2	<0.001
SBF	0.3	1	0.3	0.1	0.907
Region × SBF	7.9	1	7.9	0.3	0.568
Error	1818.3	76	23.9		
